# Induction of Endoplasmic Reticulum Stress Pathway by Green Tea Epigallocatechin-3-Gallate (EGCG) in Colorectal Cancer Cells: Activation of PERK/p-eIF2*α*/ATF4 and IRE1*α*

**DOI:** 10.1155/2019/3480569

**Published:** 2019-12-14

**Authors:** Zarith Nameyrra Md Nesran, Nurul Husna Shafie, Amirah Haziyah Ishak, Norhaizan Mohd Esa, Amin Ismail, Siti Farah Md Tohid

**Affiliations:** ^1^Department of Nutrition and Dietetics, Faculty of Medicine and Health Sciences, Universiti Putra Malaysia, 43400 UPM Serdang, Selangor, Malaysia; ^2^Laboratory of UPM-MAKNA Cancer Research, Institute of Bioscience, Universiti Putra Malaysia, 43400 Serdang, Selangor, Malaysia; ^3^Department of Biomedical Sciences, Faculty of Medicine and Health Sciences, Universiti Putra Malaysia, 43400 UPM Serdang, Selangor, Malaysia

## Abstract

Epigallocatechin-3-gallate (EGCG) is the most abundant bioactive polyphenolic compound among the green tea constituents and has been identified as a potential anticancer agent in colorectal cancer (CRC) studies. This study was aimed to determine the mechanism of actions of EGCG when targeting the endoplasmic reticulum (ER) stress pathway in CRC. The MTT (3-(4,5-dimethylthiazol-2-yl)-2,5-diphenyl tetrazolium bromide) assay was performed on HT-29 cell line and normal cell line (3T3) to determine the EGCG toxicity. Next, western blot was done to observe the expression of the related proteins for the ER stress pathway. The Caspase 3/7 assay was performed to determine the apoptosis induced by EGCG. The results demonstrated that EGCG treatment was toxic to the HT-29 cell line. EGCG induced ER stress in HT-29 by upregulating immunoglobulin-binding (BiP), PKR-like endoplasmic reticulum kinase (PERK), phosphorylation of eukaryotic initiation factor 2 alpha subunit (eIF2*α*), activating transcription 4 (ATF4), and inositol-requiring kinase 1 alpha (IRE1*α*). Apoptosis was induced in HT-29 cells after the EGCG treatment, as shown by the Caspase 3/7 activity. This study indicates that green tea EGCG has the potential to inhibit colorectal cancer cells through the induction of ER stress.

## 1. Introduction

**Figure 1:**
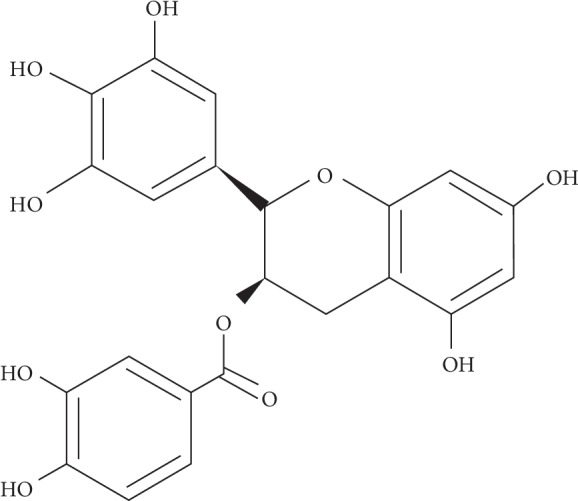
Chemical structure of EGCG compound (ChemSketch Software, Advanced Chemistry Development Labs).

Green tea has long been a part of human life, and its earliest consumption can be dated back from 500,000 years ago. Asian ancestors, particularly from China, Japan, India, and Thailand, had been using green tea for healing purposes [1]. There are studies showing the antiproliferative, antimutagenic, antioxidant, antibacterial, antiviral, anticancer, and chemopreventive effects of green tea [2, 3]. From previous studies, it is found that epigallocatechin-3-gallate (EGCG) (Figure 1) is the most bioactive and abundant polyphenolic compound in green tea [3, 4].

EGCG has always achieved a milestone in the quest for cancer therapy. It has been observed to suppress breast cancer [5–7], prostate cancer [8–10], lung cancer [11–13], pancreatic cancer [14–16], and liver cancer [17, 18]. All of this anticancer activity by EGCG has previously been demonstrated that EGCG is the most effective cancer chemopreventive polyphenol in green tea [19]. Rady et al. reviewed that all of these anticancer effects by EGCG work by apoptosis induction, control in cell proliferation, and/or inhibition of angiogenesis [20]. These mechanisms have previously been suggested by Min and Kwon, and they also added inhibition in metastasis; tumorigenesis is also an important mechanism adapted by EGCG to unveil their anticancer properties [21].

The therapeutic effects of EGCG are indeed unexceptional in colorectal cancer studies too. It has been shown to work in several mechanisms: inhibition of stem cells of colorectal cancer (CRC) via suppression of the Wnt/*β*-catenin pathway [22], impeding CRC sphere formation [23], inhibition of CRC proliferation [24], inhibition of VEGF signaling [25], and degradation of proteins [26]. The possible molecular mechanisms of EGCG in CRC involve various molecules and signaling pathways [27].

Targeting endoplasmic reticulum (ER) stress and its rescue system and unfolded protein response (UPR) is a promising field for cancer treatments. The UPR system is mediated by three key mediators, namely, pancreatic ER kinase- (PKR-) like ER kinase (PERK), inositol-requiring enzyme 1*α* (IRE1*α*), and activating transcription factor 6 (ATF6), where all are located in the membranes of ER [28]. In a resting mode, these three transmembrane proteins bind to the main chaperone, immunoglobulin-binding protein (BiP) [29]. Following an ER stress, these proteins will dissociate from BiP and activate their respective downstream cascades system [30]. The upregulation of UPR sensor proteins is often regarded as the indicator of the incidence of ER stress in cancer [31]. Prolonged ER stress may cause dysfunctional UPR, which leads cells to enter the death mode [32].

The increase in fatal rates due to colorectal cancer (CRC) is now affecting the world's population. Despite numerous studies had successfully demonstrated EGCG as an anticancer agent, none of these studies showed a clear understanding of the role of EGCG in the ER stress pathway, particularly in colorectal cancer. This study was primarily performed to introduce EGCG in combating CRC and as a safer alternative to the chemotherapy that is continuously posing side effects to the CRC patients. Hence, this study was done in order to elucidate the mechanism of EGCG actions in colorectal cancer cells via ER stress pathways.

## 2. Materials and Methods

### 2.1. Cell Culture

Cancer cell line used throughout this study was the human colorectal adenocarcinoma cell line (HT-29). The cells were grown in Dulbecco's Modified Eagle Medium (DMEM, 1X high glucose—Life Technologies, CA, USA) supplemented with 10% fetal bovine serum, 1% sodium pyruvate, and 1% penicillin-streptomycin antibiotics. The cells were maintained in an incubator with the setting of 5% CO_2_/95%O_2_ at 37°C. Meanwhile, normal cell line used was Embryonic Fibroblast Cell Line (designated as 3T3), also originated from ATCC. These cells were grown in similar supplementation as HT-29.

### 2.2. Cytotoxicity Assay

The 3-(4,5-dimethylthiazol-2-yl)-2,5-diphenyl tetrazolium bromide (MTT) assay has been performed to assess cell proliferation activity and cytotoxicity in HT-29 cells and 3T3 cells, when treated with different concentrations of EGCG extracted from green tea (Cat. #: E4143, Sigma, Missouri USA). Firstly, cells were seeded on 96-well plates overnight. After achieving confluency around 70%–80%, the HT-29 cells were treated with EGCG from 0 to 1000 (0, 125, 250, 500, and 1000) *μ*M for 24 h, 48 h, and 72 h incubation periods; meanwhile, 3T3 cells were treated with EGCG for 72 h incubation period.

### 2.3. Cell Treatments for Protein Expression Analysis

HT-29 cells were first seeded on 30 mm tissue culture treated Petri dish at 8 × 10^5^ overnight. The cells were then treated with EGCG for 24 h, 48 h, and 72 h incubation period. Protein harvesting was performed once each treatment period ended.

### 2.4. Protein Extraction

RIPA lysis buffer (Merck, MO, USA) containing 1% of protease inhibitor EDTA-free (Merck, MO, USA) and 1% of phosphatase inhibitor (Merck, MO, USA) was added to each 30 mm Petri dish containing the cells. The cells were then incubated on ice for about 5 minutes. After that, the cells were gently scraped using a cell scraper and were collected into a 2 mL microtube. The cells were further incubated on ice for 15 minutes. After that, it was centrifuged (10,000 rpm) at 4°C for 10 minutes. The supernatant was then collected. The quantification of proteins was immediately performed by using the bicinchoninic acid (BCA) protein assay kit (Thermo Fisher Scientific, MA, USA).

### 2.5. Western Blot

The protein samples were separated by SDS-PAGE at 110 V for 50 minutes. Transfer of samples from gel to the PVDF membrane was done by wet transfer with a transfer buffer (1X), loaded with ice packs, at 60 V for 2 hours. After 2 hours, membranes were dried for an hour at room temperature (RT) before being blocked with 5% BSA in TBS-T buffer for 1 hour and was gently rocked on a shaker at RT. Incubation with the primary antibody was performed in TBS-T + 5% BSA overnight in 4°C chiller with a dilution of 1 : 1000. The next day, each membrane was washed three times with TBS-T for 5 minutes. The HRP-conjugated secondary antibody was diluted at 1 : 2500 in TBS-T + 5% BSA, and then the blots were incubated for 1 h at RT and washed with TBS-T [33, 34].

Antibodies used throughout this study include BiP (Cat. #: 3177 Cell Signaling Technology), PERK (Cat. #: 5683), p-eIF2*α* (Cat. #: 9721), ATF4 (Cat. #: 11815), and IRE1*α* (Cat. #: 3294), which were all purchased from Cell Signaling Technology, MA, USA, and GAPDH (Cat. #: SC25778 Santa Cruz Biotechnology, TX, USA) and anti-rabbit IgG HRP-linked (Cat. #: 7074 Cell Signaling Technology, MA, USA).

### 2.6. Protein Detection

After incubation with the secondary antibody, the membrane was washed three times. Next, Luminata™ Forte Western HRP substrate was used for protein detection. The substrate was added onto the membrane and was let to incubate at room temperature for 2 minutes. Later, the membrane was viewed in a gel doc. The band's intensity was quantified using Image Studio Lite Software, Version 5.2. All the bands were normalized with the loading control GAPDH.

### 2.7. Caspase 3/7 Assay

EGCG treatments on the cells for the Caspase 3/7 assay (Promega, WI, USA) were based on the concentrations of IC_50_ values, respectively, for each treatment period. A volume of 100 *μ*L Caspase 3/7 reagent was added to each well containing the cells. After this addition, the plate was put on a shaker at 300–500 rpm for at least 30 seconds. When the mixture was well blended, the plate was incubated at RT for 1 hour. The samples were finally measured with luminescence setting. The gain adjustment was performed prior to the luminescence reading.

### 2.8. Statistical Analysis

The protein expression and Caspase 3/7 results were analyzed using GraphPad software (San Diego, CA, USA). The independent *t*-test was applied to compare the differences in the mean between treated samples and control samples. Differences were only considered as statistically significant when *P* < 0.05.

## 3. Results

### 3.1. EGCG Inhibited Colorectal Cancer Cell Growth

**Figure 2:**
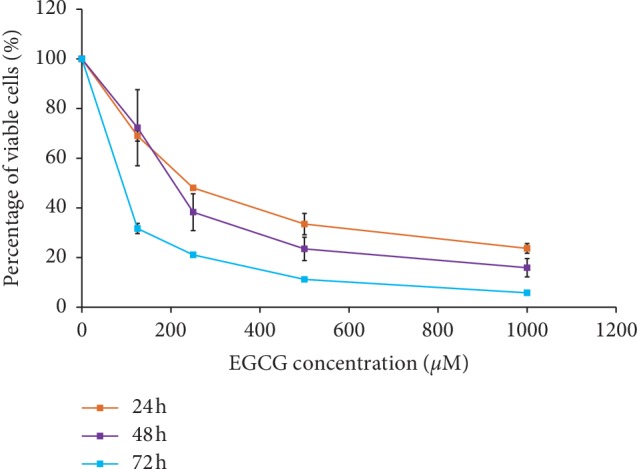
Percentage of HT-29 viable cells. EGCG treatments were given to HT-29 cells at 24 h, 48 h, and 72 h incubation times. The MTT assay was then performed at each incubation time. The results are expressed as mean percentage ± standard error of the mean (SEM) (*n*=3).

As shown in Figure 2, the number of viable cells was reduced after 24 h with the increasing concentration of EGCG. A similar observation was seen following 48 and 72 h of treatment. The viability of the cells was also affected by the duration of EGCG exposure. Thus, the toxicity of EGCG depends on the dose and duration of exposure. This clearly showed the toxicity of the EGCG towards the colorectal cancer cells in a dose-dependent manner.

The EGCG treatment does not only reduce the percentage of HT-29 viable cells by a dose-dependent manner but also works in a time-dependent manner. The comparison of the trends amongst all the incubation time concluded that the longest hour of incubation; 72 h always caused the lowest percentage of viable cells as compared to shorter treatment periods (Figure 2). The inhibitory concentrations of EGCG at 50% of HT-29 population (IC_50_) were 262.5 *μ*M, 190.3 *μ*M, and 88.1 *μ*M for 24 h, 48 h, and 72 h, respectively.

**Figure 3:**
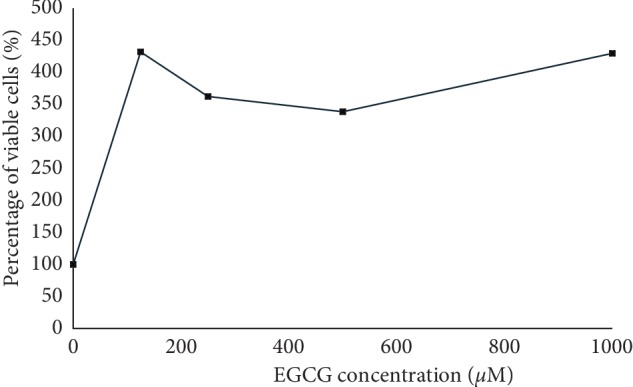
Percentage of viable cells (normal cells 3T3), given EGCG treatment at 72 h incubation time. The results are expressed as percentage ± SEM. The experiments were performed three times independently (*n*=3).

Since the study has demonstrated the toxicity of EGCG at inhibiting the growth of colorectal cancer cell lines, its toxicity has also been tested on the normal cell line, 3T3 (Figure 3). This embryonic fibroblast cell line (3T3) has shown that the EGCG was not toxic to normal healthy cells, given the treatment at any concentration even at the highest concentration of EGCG (1000 *μ*M).

### 3.2. EGCG Dissociated Chaperone Protein, BiP from UPR Protein Complexes

**Figure 4:**
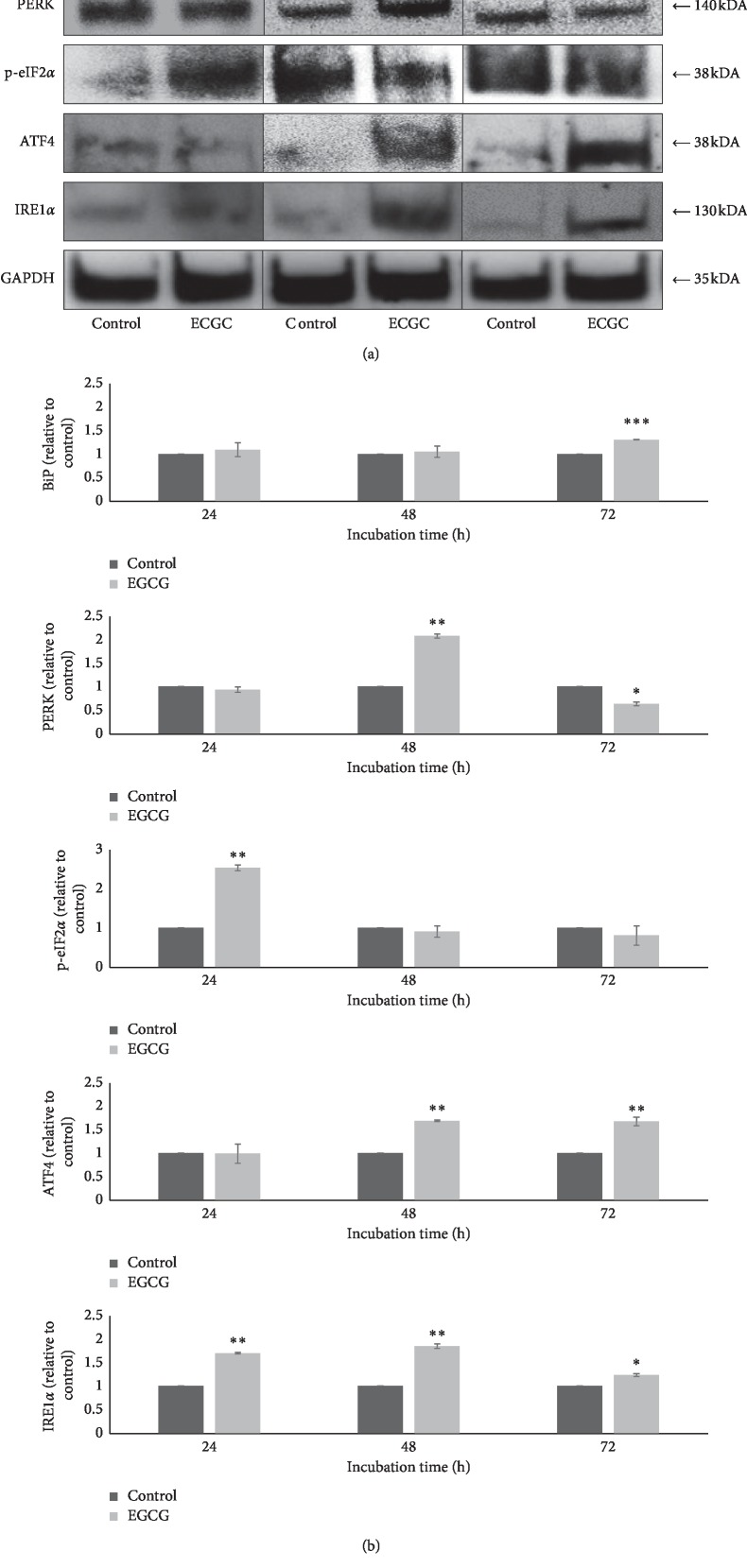
Expression of BiP, PERK, p-eIF2*α*, ATF4, and IRE1*α* after EGCG treatment. GAPDH was used as the loading control. The densitometry results are from three independent experiments and are expressed as mean ± SEM (*n*=3) normalized to GAPDH, ^*∗*^*P* < 0.05, ^*∗∗*^*P* < 0.01, and ^*∗∗∗*^*P* < 0.001, relative to their respective controls at each incubation time.

There was no significant increment of BiP expression (*P* > 0.05) following EGCG treatment after 24 and 48 h of incubation toward the HT-29 cell line (Figure 4). However, after 72 h of incubation, the expression of BiP was significantly increased (*P* < 0.001) when compared to the respective control. Hence, this indicated the occurrence of ER stress in colorectal cancer cells treated with EGCG as the expression of BiP is associated with ER stress activation.

### 3.3. EGCG Increased PERK Expression and Its Downstream Molecules p-eIF2*α* and ATF4

This study demonstrated that EGCG treatment on the HT-29 cell line has activated PERK and its downstream molecules p-eIF2*α* and ATF4. PERK expression was significantly increased (*P* < 0.01) after a 48 h incubation in EGCG-treated cells when compared to the respective control (Figure 4). However, the PERK expression was significantly decreased (*P* < 0.05) after 72 h which showed the transient expression of PERK in HT-29 cells, as shown in Figure 4.

PERK's downstream target, the phosphorylated eIF2*α* (p-eIF2*α*) expression, was significantly increased (*P* < 0.01) after EGCG treatment at 24 h of incubation when compared to the respective control (Figure 4). In addition, another downstream molecule of PERK, ATF4 expression, was significantly increased only after 48 h (*P* < 0.01) and 72 h (*P* < 0.01) of incubations with EGCG (Figure 4).

Overall, these results demonstrated that all the expressions of PERK and its downstream molecules p-eIF2*α*, and ATF4 were upregulated by EGCG. PERK induction is required for the activation of downstream UPR molecules. The activation of these molecules indicates the occurrence of ER stress in colorectal cancer cell lines, HT-29.

### 3.4. EGCG Upregulated IRE1*α* in HT-29 Cell Lines

In this study, treatment with EGCG on colorectal cancer cells (HT-29) has caused upregulation of IRE1*α* expressions at all incubation times: 24 h, 48 h, and 72 h (Figure 4). The IRE1*α* expressions were significantly increased at 24 h (*P* < 0.01), 48 h (*P* < 0.01), and 72 h (*P* < 0.05) when compared to their respective controls (Figure 4). The elevation of IRE1*α* expression was associated with the occurrence of ER stress.

### 3.5. EGCG Increased Caspase 3/7 Activity in HT-29 Cell Lines

**Figure 5:**
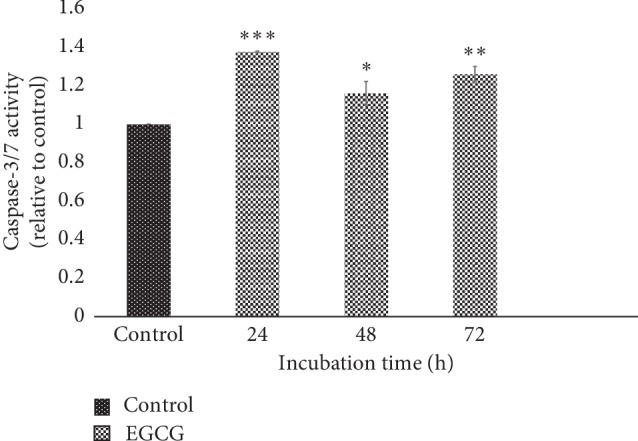
Caspase 3/7 activities at 24 h (IC_50_ = 262.5 *μ*M), 48 h (IC_50_ = 190.3 *μ*M), and 72 h (IC_50_ = 88.1 *μ*M) of incubation times. EGCG concentrations used are based on respective IC_50_ for different incubation times. Caspase 3/7 activity at different times was measured in relative to the control sample. ^*∗*^*P* < 0.05, ^*∗∗*^*P* < 0.01, and ^*∗∗∗*^*P* < 0.001.

As shown in Figure 5, treatment with EGCG for 24 h on HT-29 had significantly increased (*P* < 0.001) Caspase 3/7 activity when compared to the control. In addition, after 48 h and 72 h of incubations, the Caspase 3/7 activity was also markedly increased (Figure 5). Activated Caspase 3/7 promotes apoptosis and inhibits the normal physiological function of cancer cells. Therefore, the EGCG activated ER-stress protein and thereby induced apoptosis.

## 4. Discussion

The exploration of EGCG as an anticancer has a wide spectrum of research areas ranging from the molecular level to clinical trials. Hence, the cytotoxicity of EGCG is the first area that needs to be tackled before going further into the focused research area [35]. This study has demonstrated the toxic effect of EGCG on colorectal cancer cells similar to previous studies and does not have cytotoxic effects on normal cells.

In the prospect of colorectal cancer treatment, a number of researches have shown that EGCG absolutely does have cytotoxic effects on the colorectal cancer cells either at growth inhibition or as the cancer chemopreventive mechanism [19, 23, 24, 36]. Mechanism of chemoprevention works by preventing the establishment of cancer cells in the human body [19]. The main purpose of this study is to provide elucidation on the mechanism of actions of green tea EGCG in colorectal cancer (CRC), particularly via the endoplasmic reticulum (ER) stress pathway.

The occurrence of ER stress requires the cells to adapt to the survival mode or failure to maintain this survival which will lead the cells to undergo apoptosis [37]. The pathways activated throughout survival to cell death modes are classified under the unfolded protein response (UPR) mechanism. Due to extravagated intrinsic or extrinsic factors, these UPR pathways become markers for the severity of the ER stress [38].

Exploiting ER stress as part of cancer therapy seems a promising strategy; especially pharmacological agents are used as ER stress inducers [39]. A prolonged period of ER stress will cause cytotoxicity to the cells and hence this leads to apoptosis [40]. From this study, it was revealed that the treatment of EGCG on colorectal cancer cell lines, HT-29, had induced the expressions of UPR-related proteins which are BiP, PERK and its downstream targets (p-eIF2*α* and ATF4), and IRE1*α* (Figure 4).

Furthermore, the upregulation of BiP expressions indicates that the first step of the UPR mechanism had indeed occurred which was the dissociation of BiP from the UPR main axes. This dissociation also means that the three UPR arm proteins are expected to be activated as well [41]. This study also demonstrated the induction of ER stress by EGCG that caused marked BiP upregulation. Several pharmacological drug treatments had shown enhanced expressions of BiP which indicated the response of ER stress [38]. These studies are indeed in line with our findings in terms of BiP activation upon ER stress induction.

We had also demonstrated the activation of PERK as well as its downstream targets, p-eIF2*α* and ATF4, and IRE1*α* protein-induced ER stress after EGCG treatment. PERK and IRE1*α* have similar mechanisms of activation due to their homologous secondary structure, and both of these proteins are also dependent on the BiP association [42, 43]. Furthermore, it is anticipated that the mechanism of ER stress sensors is specifically adapted to favor the particular needs of the organisms [44]. Hence, it can be concluded that the mechanism of EGCG actions as the ER stress inducer in colorectal cancer cells had followed this homolog structural theory.

From this study, we also demonstrated that EGCG treatment had induced apoptosis of HT-29 cells via Caspase 3/7 activity. This study revealed that EGCG has the potential to induce robust Caspase 3/7 activity. Recently, the Caspase 3 activity induced by EGCG treatments was also observed in bladder cancer cells SW780 [45] and chondrosarcoma cells [46], as well as in diabetic mice [47], indicating that EGCG plays a role in apoptosis via Caspase 3 activity. In general, polyphenols such as EGCG induced apoptosis in a caspase-dependent manner [48–50]. However, EGCG has also shown its apoptotic activities via caspase-independent pathways [51, 52]. Various findings including our study confirmed that EGCG has apoptotic properties in cancer via both caspase-dependent and caspase-independent mechanisms. This study highlighted caspase 3/7-dependent apoptosis by EGCG in colorectal cancer cells, and to validate this finding, further studies such as caspase 3 and 7 genes knockout study should be carried out in the future. Though this study primarily showed the caspase-dependent pathway upon ER stress induction by EGCG, caspase-independent assays should also be investigated to explore the other potentials of EGCG as an ER stress inducer in colorectal cancer cells.

**Figure 6:**
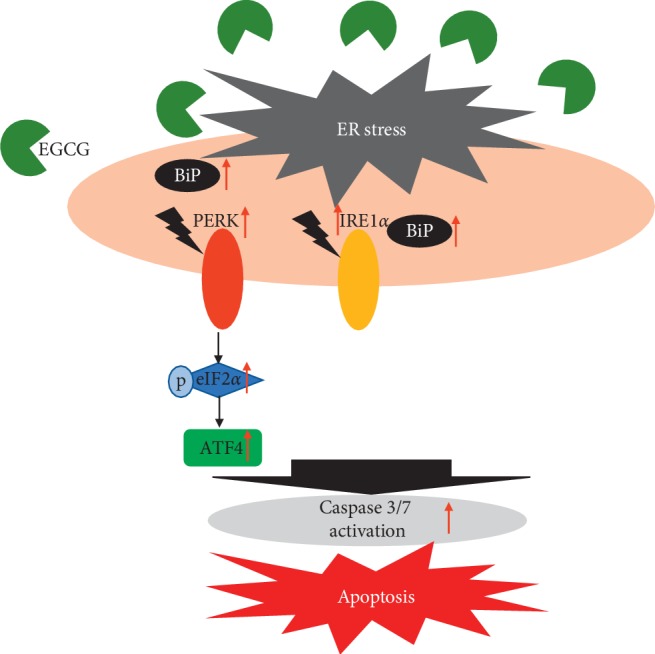
Mechanism of action of EGCG in colorectal cancer (CRC). ER stress is induced by EGCG and activates UPR proteins, PERK (with its downstream targets eIF2*α* and ATF4), and IRE1*α*. Caspase 3/7 activity is also enhanced, indicating apoptosis occurrence in the colorectal cancer cells.

As mentioned previously, prolonged or chronic ER stress and UPR activation will lead the cells to enter death mode. Based on our findings, due to the ER stress induced by EGCG, HT-29 cells had entered apoptosis mode via Caspase 3/7 activity. Furthermore, key players of cell death resulted from ER stress are dependent on UPR sensors as well. For instance, activation of PERK/p-eIF2*α*/ATF4 will initiate proapoptotic signaling pathways [53, 54]. This study demonstrated that EGCG treatment on HT-29 cells has activated PERK and its downstream molecules (p-eIF2*α* and ATF4) and IRE1*α* which can trigger ER stress-induced apoptosis. Collectively, it can be concluded that EGCG has induced ER stress in CRC which eventually leads to apoptosis.  Figure 6 briefly illustrates the mechanism of action of EGCG as an ER stress inducer in CRC.

## 5. Conclusions

From this study, the mechanism of actions of green tea EGCG in colorectal cancer was elucidated via the induction of the ER stress particularly through PERK/p-eIF2*α*/ATF4 and IRE1*α* pathways, which eventually lead to apoptosis. In addition, this present study has discovered the potential of EGCG in targeted colorectal cancer therapy. The breakthrough from this side will surely help in combating the rising of CRC incidences globally.

## Data Availability

The data used to support the findings of this study are available from the corresponding author upon request.

## Abbreviations

EGCG: Epigallocatechin-3-gallate
DFO: Desferrioxamine
CRC: Colorectal cancer
ER: Endoplasmic reticulum
MTT: 3-(4,5-Dimethylthiazol-2-yl)-2,5-diphenyl tetrazolium bromide
RT: Room temperature
BiP: Immunoglobulin-binding protein
PERK: PKR-like endoplasmic reticulum kinase
p-eIF2*α*: Phosphorylation of eukaryotic initiation factor 2 alpha subunit
ATF4: Activating transcription 4
IRE1*α*: Inositol-requiring kinase 1 alpha
UPR: Unfolded protein response
VEGF: Vascular endothelial growth factor
DMEM: Dulbecco's modified Eagle medium
RIPA: Radioimmunoprecipitation assay
EDTA: Ethylenediaminetetraacetic acid
BCA: Bicinchoninic acid
SDS-PAGE: Sodium dodecyl sulfate-polyacrylamide gel electrophoresis
TBS-T: Tris-buffered saline Tween
GAPDH: Glyceraldehyde 3-phosphate dehydrogenase.
